# Respiratory Colonization and Short-Term Temporal Changes in the Urinary Metabolome of Children

**DOI:** 10.3390/metabo11080500

**Published:** 2021-07-30

**Authors:** Lilliam Ambroggio, Todd A. Florin, Kayla Williamson, Cory Pfefferman, Brandie D. Wagner, Larisa Yeomans, Jae Hyun Kim, Heidi Sucharew, Maurizio Macaluso, Richard M. Ruddy, Samir S. Shah, Kathleen A. Stringer

**Affiliations:** 1 Sections of Emergency Medicine and Hospital Medicine, Children’s Hospital Colorado & Department of Pediatrics, University of Colorado, Aurora, CO 80045, USA; 2 Division of Emergency Medicine, Ann and Robert H. Lurie Children’s Hospital of Chicago & Department of Pediatrics, Feinberg School of Medicine, Northwestern University, Chicago, IL 60611, USA; taflorin@luriechildrens.org; 3 Department of Biostatistics and Informatics, Colorado School of Public Health, University of Colorado, Aurora, CO 80045, USA; kayla.3.williamson@cuanschutz.edu (K.W.); brandie.wagner@cuanschutz.edu (B.D.W.); 4 Division of Biostatistics and Epidemiology, Cincinnati Children’s Hospital Medical Center & Department of Pediatrics, College of Medicine, University of Cincinnati, Cincinnati, OH 45267, USA; cory.pfefferman@cchmc.org (C.P.); heidi.sucharew@cchmc.org (H.S.); Maurizio.Macaluso@cchmc.org (M.M.); 5 Nuclear Magnetic Resonance Metabolomics Laboratory, Division of Pulmonary and Critical Care Medicine, Department of Clinical Pharmacy, College of Pharmacy, Michigan Center for Integrative Research in Critical Care, School of Medicine, University of Michigan, Ann Arbor, MI 48109, USA; yeomans@umich.edu (L.Y.); kimjae@umich.edu (J.H.K.); stringek@med.umich.edu (K.A.S.); 6 Division of Emergency Medicine, Cincinnati Children’s Hospital Medical Center & Department of Pediatrics, College of Medicine, University of Cincinnati, Cincinnati, OH 45267, USA; richard.ruddy@cchmc.org; 7 Divisions of Hospital Medicine and Infectious Diseases, Cincinnati Children’s Hospital Medical Center & Department of Pediatrics, College of Medicine, University of Cincinnati, Cincinnati, OH 45267, USA; samir.shah@cchmc.org

**Keywords:** pediatric, healthy, metabolome, nuclear magnetic resonance, *Streptococcus pneumoniae*, rhinovirus/enterovirus

## Abstract

The human metabolome may vary based on age, over time, and in the presence of viral carriage and bacterial colonization—a common scenario in children. We used nuclear magnetic resonance spectroscopy to identify and quantify urinary metabolites of children without signs or symptoms of respiratory illness. A urine sample and two nasopharyngeal swabs were collected to test for respiratory viral pathogens and colonization by *Streptococcus pneumoniae* (*Sp*). Urine samples were collected at the initial visit, 24 h post-enrollment, and 10–14 days post-enrollment. Of the 122 children enrolled, 24% had a virus detected and 19.7% had *Sp* detected. Intraclass correlation coefficients demonstrated greater within-subject versus between-subject variability for all metabolites detected. In linear mixed models adjusted for age, time, history of asthma, *Sp*, and viruses, 1-methylnicotinamide was increased by 50% in children with *Sp* and decreased by 35% in children with rhinovirus/enterovirus. Children with *Sp* had 83% higher levels of trimethylamine-N-oxide compared with those without *Sp*. However, when adjusting for multiple comparisons, the association was no longer statistically significant. In conclusion, there appear to be short-term changes within the urinary metabolome of healthy children, but levels of metabolites did not statistically differ in children with viral carriage or *Sp* detected.

## 1. Introduction

The human metabolome can be affected by environmental (e.g., diet, medications) and biological (e.g., age, sex) factors. In adults, metabolites that differentiate sex are mostly the result of changes in energy metabolism (e.g., creatine), as men have more muscle mass than women [1,2]. In prepubescent, non-neonate children, the difference in muscle mass is much less pronounced, making the impact of sex less than the impact of age alone [3]. Studies assessing the impact of diet on the metabolome in adults have shown greater variability in the metabolome between subjects than within subjects [4,5], but such variability did not distinguish healthy and non-healthy subjects [5]. Age may be an important predictor of metabolite changes over time in adults [1,4]. In children, the metabolome also varies with age [6]; however, acute changes over time in the metabolome of children remain of interest.

The nasopharynx of healthy children is likely to be colonized with commensal bacteria, such as *Streptococcus pneumoniae (Sp)* [7]. In up to 6% of children, *Sp* can become pathogenic, and cause significant morbidity, such as pneumonia [8,9]. Children are also commonly asymptomatic viral carriers and can shed viruses for prolonged periods. There is limited information about the association between a child’s metabolome and bacterial colonization and being a viral carrier. To draw reliable conclusions about the metabolomic profiles of respiratory diseases in children, investigating *Sp,* viral carriage, and their influence on metabolomes in asymptomatic children is essential. 

The urinary metabolome of children is ideal for studying metabolic changes, viral carriage, and bacterial colonization over a short time, as it is easily accessible and provides a comprehensive view of the child’s physiology. The objectives of this study were to (1) determine within-subject and between-subject variation in the urinary metabolomes of children over a short time period, and (2) to identify urinary metabolites associated with *Sp* colonization and viral carriage in the nasopharynx of healthy children without respiratory signs or symptoms. 

## 2. Results

### 2.1. Study Population Characteristics 

The study included 122 children who provided three urine samples, and had complete data available, out of a total of 185 enrolled. Most of the children (76, 62.3%) were between 5 and 12 years of age, and 11 (9%) had a history of asthma (Table 1). No cigarette smoke exposure was reported for any of the participants. Although 83.6% of the children had a biological sibling enrolled in the study, no statistical association was found between metabolite levels within families. Prior to enrollment, 68 (55.7%) of the children reported having received a vaccination against influenza during the most recent flu season (Table 1).

Although all of the children were asymptomatic, 24 (19.7%) had a detectable load of *Streptococcus pneumoniae* (*Sp*) in their nasopharynx, determined by the presence of *Sp* autolysin A (lytA) (Table 1). Among those with *Sp*, the median load was 24,643 copies/mL (interquartile range: 3174–496,021). The most frequently detected virus in the nasopharynx was rhinovirus/enterovirus (21, 72.4%), with parainfluenza and coronavirus each detected in 3 (10.3%) of the children.

### 2.2. Temporal Variation in the Urinary Metabolome 

**Figure 1:**
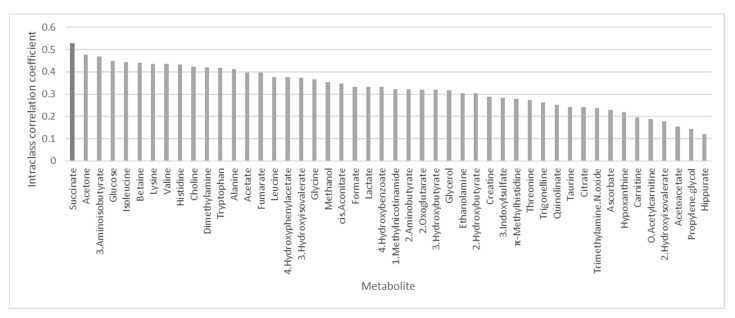
Intraclass correlation coefficient (ICC) for each metabolite ordered from the largest (succinate) to the smallest ICC (hippurate). ICC estimates of the proportion of the total variability that is due to between-subject variability. An ICC above 0.5 (bold line) indicates higher similarity between measurements across children. ICC values below 0.5 (grey line) indicate higher similarity for the measurements within children. Most metabolites have higher variation within children than across children.

Of the 51 NMR-detected, creatinine-normalized metabolites, three metabolites (fructose, sucrose, and 1,6-Anhydro-β-d-glucose) had greater than 90% missing values across samples and were excluded from the analysis. We compared the metabolite levels across the three timepoints (Table S1). There were no statistically significant changes in any of the metabolites across the three timepoints. The metabolites with the highest intraclass correlation (ICC) included succinate (0.528), acetone (0.477), and 3-aminoisoutyrate (0.470) (Figure 1, Table S2). Most metabolites, except succinate, had ICCs below 0.5, indicating that there was higher within-subject variability than between-subject variability (i.e., values from the same subject were more variable across the timepoints than values across subjects and time). 

**Figure 2:**
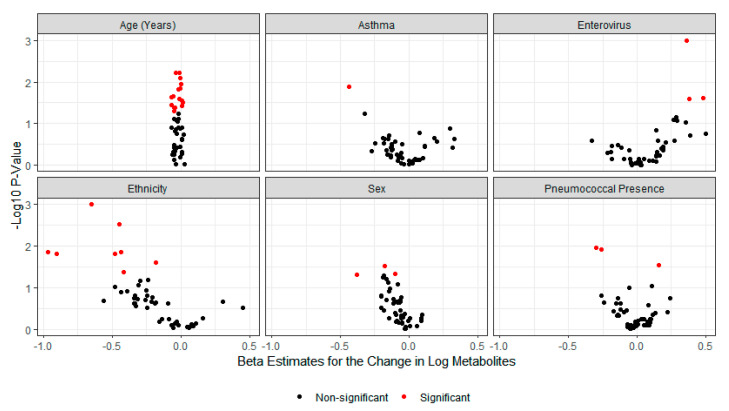
Each plot shows the -log_10_ of the nominal *p*-value on the *y*-axis and the log_10_ fold change in metabolite concentration on the *x*-axis for the 122 children. Red points toward the top left represent statistically significant (*p* < 0.05) negative fold changes in metabolite concentrations, while red points toward the top right represent statistically significant positive fold changes in metabolite concentrations for each variable. Metabolites associated with age include valine, lysine, formate, creatine, 2-aminobutyrate, 2-oxoglutarate, tryptophan, betaine, quinolinate, hydroxyisovalerate, and hypoxanthine. 1-Methylnicotinamide was statistically associated with asthma. Quinolinate, 3-indoxylsulfate, and citrate were statistically associated with rhinovirus/enterovirus. Ethnicity was statistically associated with alanine, tryptophan, propylene glycol, threonine, betaine, histidine, ethanolamine, and 2-oxoglutarate. Sex was associated with cis-aconitate, threonine, and propylene glycol. Pneumococcal presence was associated with TMAO, hippurate, and citrate.

**Figure 3:**
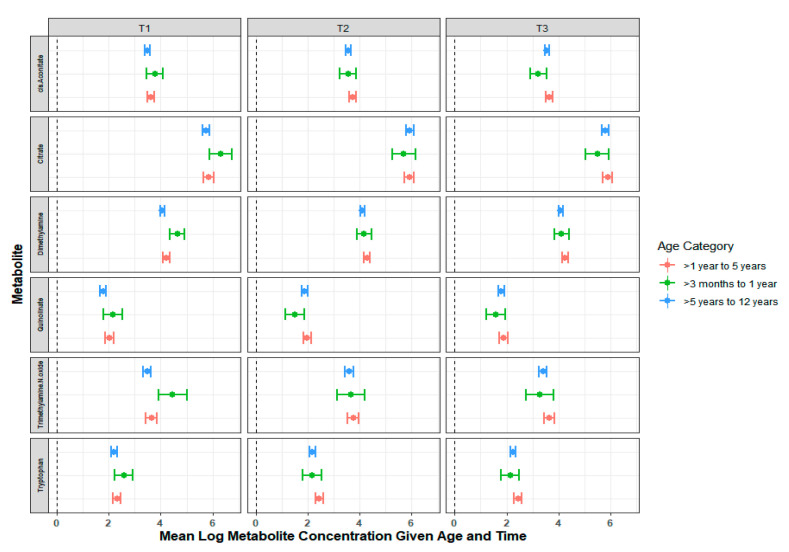
Least square means and corresponding 95% confidence intervals of the log values for the metabolites (concentration in µM) with a significant age–time interaction from the linear mixed model. Each column presents a unique time point (T1 indicating the initial visit, T2 indicating 24 h after the initial visit, and T3 indicating 10–14 days after the initial visit); metabolites are presented in rows. Children aged >3 months to 1 year represent 5.7% (*n* = 7), >1 year to 5 years represent 32% (*n* = 39), and >5 to 11 years old represent 62.3% (*n* = 76) of the total study cohort. Each colored data point represents a different age category.

Metabolites were tested for their association with age, asthma history, rhinovirus/enterovirus, ethnicity, sex, and *Sp* presence (Figure 2). Metabolites associated with age included valine, lysine, formate, creatine, 2-aminobutyrate, 2-oxoglutarate, tryptophan, betaine, quinolinate, hydroxyisovalerate, and hypoxanthine. 1-Methylnicotinamide was statistically associated with asthma. Quinolinate, 3-indoxylsulfate, and citrate were statistically associated with rhinovirus/enterovirus. Ethnicity was statistically associated with alanine, tryptophan, propylene glycol, threonine, betaine, histidine, ethanolamine, and 2-oxoglutarate. Sex was associated with cis-aconitate, threonine, and propylene glycol. Pneumococcal presence was associated with TMAO, hippurate, and citrate. Using a linear mixed model controlling for repeated measures, the interaction between age and time was investigated for metabolites (Figure 3). Tryptophan, trimethyl-N-oxide (TMAO), quinolinate, dimethylamine, citrate, and cis-aconitate had differential association for time depending on their age category (Figure 3). Only three metabolites—1-methylnicotinamide, 3-hydroxybutyrate, and TMAO—had a significant global test for a model that contained age, time, asthma, *Sp* presence, virus, and an age–time interaction term (Table 2). None of the global tests for any of the metabolites remained statistically significant after adjusting for multiple comparisons (Table S3).

### 2.3. Influence of Bacterial Colonization and Viral Carriage on the Urinary Metabolome 

Children with rhinovirus/enterovirus had, on average, 34.9% lower levels of 1-methylnicotinamide than those without, holding all other variables constant. Children with *Sp* had, on average, 50.4% higher levels of 1-methylnicotinamide than those without. Age and asthma history were not statistically associated with 1-methylnicotinamide (Table 2). Children with presence of *Sp* had, on average, 83.1% higher levels of trimethyl-n-oxide, when compared with those without (Table 2). However, age, time, asthma, rhinovirus/enterovirus, and the interaction between age and time were not statistically associated with trimethyl-*n*-oxide. 3-Hydroxybutyrate was not statistically associated with any individual variable in the adjusted model.

## 3. Discussion

We found that among a cohort of healthy children with no signs or symptoms of respiratory illness, 24% had a virus detected and 19.7% had *Sp* detected. Metabolite levels did not change over time when accounting for multiple comparison testing. We also found that propylene glycol may have been associated univariately with ethnicity; however, it was no longer statically associated once adjusting for other variables. Additionally, propylene glycol is an exogenous metabolite and may reflect environmental exposure, as it is a common ingredient in many topical lotions, such as sunscreen. Unlike in previous studies, diet in younger children—such as breast-fed versus formula-fed—was not statistically associated with any metabolites when tested univariately in our study. This could be due to the small number of children < 2 years old (*n* = 12). Metabolites that were statistically associated with *Sp* included 1-methylnicotinamide and TMAO. Only 1-methylnicotinamide was associated with the presence of rhinovirus/enterovirus. However, these associations did not survive multiple comparison adjustment. The low ICC values for all metabolites suggested a high within-person variance, indicating a low reliability or less stable fluctuations across time within the same child. Therefore, a single measurement in time may not be appropriate in children when investigating a response to therapy but may be useful in determining disease versus health at a single timepoint. 

Older age was associated with six metabolites, two of which were tryptophan—an amino acid—and quinolinate—a metabolite of tryptophan—which commonly fluctuate due to diet [10]. TMAO can be found in normal diets when subjects eat high levels of seafood, but it has often been attributed to the gut microbiome—specifically to bacteria such as *Bacteroidetes* and *Ruminococcus* [11]. The pediatric gut microbiome is dominated by *Bacteroidetes*, and becomes less diverse with increasing age, which may also explain the increased levels of TMAO in older children compared with younger children [12]. Cis-aconitate and citrate—both part of the tricarboxylic acid (TCA) cycle—are involved in energy regulation [10]. Unlike previous studies, which investigated changes in the urinary metabolome across years, we investigated a much shorter period of time, over days. One study that investigated a birth cohort of children from 6 months to 4 years found that glycine and glutamine levels declined after 6 months of age, whereas TMAO and betaine had the highest levels in children 6 months of age when compared with other age groups [6]. Additionally, age has been associated with decreases in creatinine, glycine, betaine, citrate, succinate, and acetone, and increases in creatine [13,14]. In a large, multicenter, cross-sectional study of children from six European countries, urinary creatinine was the only urine metabolite associated with age [14]. Changes in the urinary metabolome of children seem to be larger across years—during which children experience physiological changes—than across days (i.e., age group). 

We found that only two metabolites—TMAO and 1-methylnicotinamide—were potentially associated with *Sp* detection in children. In *Sp*-infected mice, 20 metabolites were found to be associated with infection, specifically related to the TCA-cycle (e.g., 2-oxoglutarate, citrate, and succinate), gut microbiome (e.g., hippurate and TMAO), and others (e.g., betaine and trigonelline) [15]. Although we similarly found TMAO to be associated with *Sp* detection, the concentration of TMAO increased in children with *Sp* colonization, in contrast to the decrease found in mice with *Sp* infection. The same study also found that 1-methylnicotinamide, which we found was associated with *Sp* detection, was also associated with methicillin-resistant *Staphylococcus aureus*. However, we did not test for MRSA, and are thus unable to verify this result in children. We also found that 1-methylnicotinamide may be associated with the presence of rhinovirus/enterovirus after adjusting for other clinical factors, but this association was not statistically different when compared with the null model. This metabolite is part of the nicotinamide pathway and has anti-inflammatory properties. Another study found that lower levels of 1-methylnictoniamide are present in children with asthma [16]. However, the overall models showed that these changes in metabolites were not statistically different to normal diurnal fluctuation in the urinary metabolome in children. 

There are several limitations to this study: First, we standardized all metabolites by urine creatinine concentration, as is common practice for urinary metabolomics analyses. Previous literature suggests that adjusting for creatinine could artificially create correlation with age [10]. However, we found no statistical correlation between age and creatinine levels. Additionally, creatinine adjustment may not be appropriate when the child presents with substantial metabolic dysregulation, such as in the presence of infection [17]. However, our population had no signs or symptoms of metabolic dysregulation. Second, we did not have the children or their families complete food diaries to determine the association between changes in metabolite levels and changes in diet over the three timepoints. However, a previous study showed that metabolites changed more with age than with diet alone [6]. In addition, metabolites associated with the TCA cycle and oxidative phosphorylation show a strong correlation with changes in the gut microbiome. Healthy children are less likely to produce as strong of an inflammatory response when compared with healthy adults, as their microbiomes are richer and more complex than those of adults [12]. Third, our study recruited healthy children from one geographic area; thus, their urinary metabolomes may not be representative of healthy children in different geographical areas, and with different environmental exposures. Lastly, we were unable to validate our findings in an external cohort; therefore, our results should be interpreted with caution. Additional external validation of our results is important in either confirming or disputing our findings. 

In summary, our study highlights the importance of considering the overall age group in years across a cohort of children that are known to be colonized with bacteria or carry viruses. Here, we demonstrate that despite verified bacterial and viral colonization, the urinary metabolome was not systematically affected by these factors. Furthermore, we observed that the metabolome in children is highly variable within subjects, especially within the youngest age group, necessitating the inclusion of repeated measurements in future studies when investigating long-term changes (e.g., in response to therapy), in order to adequately quantify the noise and distinguish it from true signal. 

## 4. Materials and Methods 

### 4.1. Study Design and Study Subjects

This was a longitudinal study of healthy children ranging in age from 3 months to 12 years old. Children and their guardians (parents or legal guardians) were recruited through electronic mail and social media outlets associated with the Schubert Research Clinic at the Cincinnati Children’s Hospital Medical Center. Recruitment occurred from April 2017 to February 2018 to capture children during one respiratory and one non-respiratory season. Children were excluded from the study if they were hospitalized 14 days prior to their visit, were immunodeficient or immunosuppressed, had a chronic pulmonary disease (except for asthma), chronic cardiac disease, sickle cell disease, neuromuscular disorder, or any history of aspiration or aspiration pneumonia.

Participants received USD 20 as compensation for completing all of the study procedures. All guardians gave their informed consent for inclusion of their child, and assent was obtained in children >11 years, before they participated in the study. The study was conducted in accordance with the Declaration of Helsinki, and the protocol was approved as research on human subjects by the Ethics Committee of Cincinnati Children’s Hospital Medical Center (Protocol #2014-1390)

### 4.2. Study Definitions

Demographics and clinical data were prospectively obtained from the parent or legal guardian of the child. Demographics included age, sex, race, ethnicity, mother’s education, breast feeding for children <2 years old, and smoking status of the parents or legal guardians, along with the child’s exposure to smoke from other sources (i.e., exposure in the car or at any other location other than the home). Clinical data included history of asthma, history of pneumonia, prescription or over-the-counter medications the child was currently taking, immunization status (i.e., up-to-date or not), seasonal flu vaccination status, any current respiratory symptoms (e.g., fever, cough, wheezing, etc.), and whether any medical care visits were sought within the timeframe of the study, along with the reason for the visit. We did not obtain data on muscle mass, height, or weight, and are therefore unable to comment on the effects of BMI or muscle mass on the metabolome. The clinical data were obtained to ensure that the children were healthy and had no respiratory symptoms at the time of urine sample collection. Additionally, data were collected on the inclusion of children from the same family, although no more than two children from the same family were eligible for inclusion.

### 4.3. Sample Collection

Urine samples were collected at three timepoints from each participant: at the initial enrollment, 24 h post-enrollment, and 10–14 days post-enrollment. For the initial enrollment sample, older children were given urine cups directly for collection. For younger children, cotton balls were placed in the child’s diaper. Urine-soaked cotton balls were then placed in a syringe provided by the study, and urine was extracted into a urine cup for further processing and storage. Prior to enrollment, we analyzed the cotton balls, urine collection cup, and syringe used via NMR. We found that the cotton balls produced negligible levels of glycerol and acetone. The urine collection cup and syringe had no metabolite contaminates. We found no age-related association with glycerol and acetone, and therefore did not exclude any samples from the younger children. Participants were given a home urine collection kit during enrollment, which included specific instructions regarding the collection and dropping off or mailing of the second and third urine samples, two pre-coated urine cups, two ice packets, an insulated mailing box, a shipping label, and two temperature strips to determine the amount of time for which the sample was at any temperature above 4 °C. Urine samples were collected at a single timepoint during the day, and were not pooled samples over the 24-h period. Urine samples were collected in urine collection cups coated with 10% sodium azide solution in order to prevent additional bacterial growth [18]. Once urine samples were received, they were centrifuged for 10 min, and the supernatant was then aliquoted into 1-mL cryovials. The samples were then frozen at −80 °C until assayed. 

At the time of enrollment, two nasopharyngeal (NP) swabs were also collected. NP swabs were placed in viral transport medium and skimmed milk–tryptone–glucose–glycerin (STGG) [19], and then frozen at −80 °C. The NP swabs in the viral transport medium were assayed using the NxTAG Respiratory Pathogen Panel kit on the MAGPIX Instrument (a Luminex platform). The assay detects influenza A (i.e., H1 subtype, 2009 H1N1 subtype, H3 subtype), influenza B, RSVA, RSVB, coronavirus (229E, OC43, NL63, HKU1), human metapneumovirus, rhinovirus/enterovirus, adenovirus, parainfluenza (1, 2, and 3), human bocavirus, *Chlamydophila pneumoniae*, *Mycoplasma pneumoniae*, and *Legionella pneumophila*. All viral testing was qualitative; therefore, only the presence or absence of the viruses was reported. The NP swab in STGG medium was tested for *Sp* autolysin A (lytA), a gene fragment that indicates the presence of *Sp* [20]. *Sp* load was quantitative; therefore, a value for each specimen was reported unless no *Sp* load was detected. We examined the load both as a continuous variable, and with the variable dichotomized as present (>500 copies/mL) or absent (≤500 copies/mL), based on the level of detection of the assay.

### 4.4. Urine Metabolite NMR Measurments

At the time of the assay, samples were thawed at room temperature. Samples were randomly batched and processed to minimize artificial effects that may arise due to day-to-day variation in NMR runs. The pH of each sample was adjusted with NaOH or HCl to achieve a pH of 7.0 ± 0.25 [21]. A volume equivalent to 10% of the total sample volume of 5.4 mM 4,4-dimethyl-4-silapentane-1-sulfonic acid (DSS-d6) in D_2_O was added to each sample as an internal standard. A one-dimensional ^1^H-NMR spectrum of each urine sample was acquired using the first increment of the standard NOESY pulse sequence on a Bruker 600 MHz spectrometer at the Cincinnati Children’s Hospital Medical Center [1,15,21]. All spectra were recorded at 25 °C. Spectral analysis was performed using quantitative metabolomics [22,23]. The raw ^1^H-NMR spectra were processed using Chenomx software (NMR Suite 7.5; Chenomx Inc., Edmonton, AB, Canada) [1,21]. NMR spectral analysis was completed by two investigators (L.Y. and J.H.K.), with routine quality checks. The resulting quantified metabolites were standardized to urinary creatinine by dividing the concentration of each metabolite by the associated creatinine concentration, as determined from the NMR spectrum of the same sample [10,24].

### 4.5. Statistical Analysis 

Continuous variables are presented as median and IQR, and categorical variables are presented as counts and percentages. The quantified metabolite data were log transformed to achieve a normal distribution of the data to conduct parametric statistical analysis [21,25]. Only metabolites that were reported in at least 90% of the samples were included in the analysis. For other metabolites, if levels were undetected or missing, a complete case analysis was performed. For each metabolite, we estimated the between-subject and within-subject variability by using linear mixed models. Metabolite concentration was the dependent variable, and a random effect was included for subject and time nested within subject. The covariance matrix for the random effects was specified to be compound symmetric [26,27]. Using these variance components, we estimated the intraclass correlation (ICC), along with the ratio of between-subject variance and total variance. Thus, a low ICC indicates that there is more variability in the metabolome within a subject than between subjects. We examined the associations with age, time, asthma, *Sp* presence, viral detection, and age–time interaction by including these variables as fixed effects in the linear mixed models. To account for multiple testing for the multiple metabolite outcomes, we used the Benjamini–Hochberg false discovery rate procedure [28]. All tests were conducted in R version 4.0.4 (2021-02-15) (R Core Team 2020). GGplot2 was used to create all graphics [29]. ICC and random effects models were created using the lme4 and lmerTest packages [30,31].

## Figures and Tables

**Table 1:** Characteristics of the study participants.

Characteristic	All (*n* = 122)
**Age Category**	
>3 Months to 1 Year	7 (5.7%)
>1 Year to 5 Years	39 (32.0%)
>5 Years to 12 Years	76 (62.3%)
**Male (Sex)**	72 (59.0%)
**Race**	
White	89 (73%)
African American	23 (19%)
Other	10 (8%)
**Ethnicity**	
Hispanic	6 (5.0%)
Not Hispanic	115 (95.0%)
**Breast Fed (*n* = 12)**	
Exclusively Breast-Fed	9 (75.0%)
Exclusively Formula-Fed	2 (16.7%)
Mixed	1 (8.3%)
**Sample also Obtained from Biological Sibling**	102 (83.6%)
**Mother’s Education**	
High School or Less	8 (6.6%)
Some College or Associates	31 (25.4%)
Bachelors	26 (21.3%)
Masters	43 (35.2%)
Professional or Doctoral	14 (11.5%)
**Asthma History**	11 (9.0%)
**Seasonal Flu Vaccine Received**	68 (55.7%)
**No Virus Detected**	93 (76.0%)
**Virus Detected**	29 (24.0%)
Bocavirus	1 (3.4%)
Coronavirus	3 (10.3%)
Influenza	1 (3.4%)
Rhinovirus/Enterovirus	21 (72.4%)
Parainfluenza	3 (10.3%)
*Sp* Load (copies/mL) *	24,643 (3174, 496,021)
***Sp* Presence**	
Positive	24 (19.7%)
Negative **	98 (80.3%)

* *Sp* load is presented for loads above the level of detection (500 copies/mL). ** Negative corresponds to ≤500 copies/mL.

**Table 2:** Estimates from linear mixed models for each metabolite with a significant likelihood ratio test (LRT) comparing the full model to a null model with no covariates (global test). Metabolite levels were transformed on the log e scale to generate a normal distribution. Both unadjusted and BH-adjusted *p*-values for the LRT are shown. Independent variables in the full model included age, time, asthma diagnosis, *Sp* presence, viruses, age–time interaction term, and a random effect for subject and time within subject.

Metabolite	Predictor	Beta Estimate	Standard Error	% Change	*p*-Value	LRT *p*-Value	BH Corrected LRT *p*-Value
1-Methylnicotinamide	(Intercept)	2.58	0.33	1219.53	<0.01	0.02	0.52
	Age (years)	−0.01	0.06	−1.149	0.84		
	Time	−0.07	0.14	−7.048	0.61		
	Asthma	−0.19	0.23	−16.88	0.43		
	*Sp* Presence	0.41	0.15	50.349	0.01		
	Rhinovirus/Enterovirus	−0.43	0.16	−34.875	0.02		
	Age–Time Interaction	−0.01	0.03	−0.569	0.83		
3-Hydroxybutyrate	(Intercept)	2.15	0.31	758.009	<0.01	0.03	0.52
	Age (years)	−0.06	0.05	−5.681	0.28		
	Time	−0.19	0.13	−17.489	0.16		
	Asthma	−0.28	0.2	−24.681	0.18		
	*Sp* Presence	0.18	0.13	20.083	0.18		
	Rhinovirus/Enterovirus	−0.18	0.15	−16.204	0.24		
	Age–Time Interaction	0.01	0.03	1.024	0.68		
Trimethylamine-n-Oxide (TMAO)	(Intercept)	4.21	0.46	6633.51	<0.01	0.04	0.52
	Age (years)	−0.13	0.08	−11.916	0.13		
	Time	−0.35	0.18	−29.622	0.06		
	Asthma	−0.52	0.35	−40.496	0.16		
	*Sp* Presence	0.61	0.23	83.097	0.02		
	Rhinovirus/Enterovirus	0	0.26	0.339	0.99		
	Age–Time Interaction	0.06	0.03	6.048	0.09		

## Data Availability

The data presented in this study are available in article and supplementary materials.

## Supplementary Materials

The following are available online at https://www.mdpi.com/article/10.3390/metabo11080500/s1, Figure S1: Stick Plots of Changes by Metabolite, Table S1: Geometric mean and standard deviation of individual metabolites at each time point, Table S2: Intraclass correlation for each metabolite, Table S3: *p*-values for the likelihood ratio test for the full adjusted model compared with the null models.
