# Supplementary material for: Respiratory Colonization and Short-Term Temporal Changes in the Urinary Metabolome of Children
Source: Metabolites. 2021 Jul 30;11(8):500. doi: 10.3390/metabo11080500 (PMC8400807; doi:10.3390/metabo11080500)
Supplement: Supplementary file 1 [file metabolites-11-00500-s001.zip › metabolites-1318796-supplementary.pdf]

Respiratory Colonization and Short-Term Temporal Changes in the Urinary Metabolome of Children

Supplementary Materials:

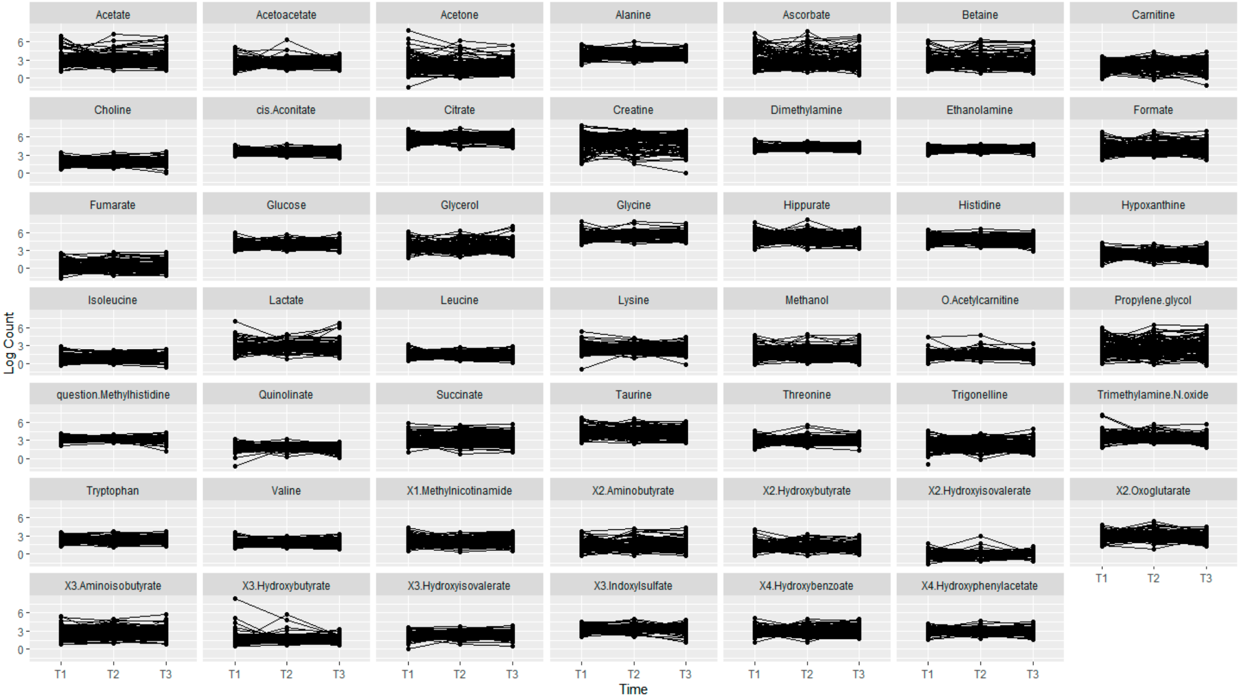

Figure S1. Stick Plots of Changes by Metabolite. Comparison of time points (x-axis) and log concentration of each metabolite (y-axis) identified from 122 healthy children (metabolites are ordered from most statistically significant changes to least statistically significant change). Each line represents an individual subject and their corresponding time points.

Table S1. Geometric mean and standard deviation of individual metabolites at each time point.

Metabolite	Time Point (Mean, Standard Deviation)			P-Value*	Adjusted P-value**
	**T1**	**T2**	**T3**		
1-Methylnicotinamide	8.62 (2.13)	8.06 (1.84)	8.31 (1.99)	0.63	0.95
2-Aminobutyrate	3.78 (2.30)	3.95 (2.48)	3.82 (2.28)	0.88	0.99
2-Hydroybutyrate	3.27 (1.93)	3.37 (2.02)	3.18 (1.87)	0.70	0.95
2-Hydroyisovalerate	0.77 (1.75)	0.80 (1.78)	0.73 (1.62)	0.31	0.88
2-Ooglutarate	17.22 (2.03)	19.68 (2.18)	15.60 (1.94)	0.02	0.36
3-Aminoisobutyrate	14.44 (2.39)	16.02 (2.39)	15.09 (2.33)	0.43	0.90
3-Hydroybutyrate	5.15 (2.44)	4.81 (2.01)	4.47 (1.65)	0.18	0.85
3-Hydroyisovalerate	9.98 (1.82)	10.10 (1.63)	9.44 (1.68)	0.40	0.90
3-Indoylsulfate	29.98 (1.72)	32.64 (1.70)	29.99 (1.78)	0.27	0.88
4-Hydroybenzoate	20.48 (1.94)	18.50 (2.02)	19.35 (2.02)	0.37	0.89
4-Hydroyphenylacetate	18.94 (1.66)	19.28 (1.63)	19.04 (1.68)	0.94	0.99
Acetate	20.09 (2.95)	21.10 (2.57)	20.94 (2.80)	0.88	0.99

Acetoacetate	12.23 (2.00)	12.77 (2.01)	12.02 (1.72)	0.72	0.95
Acetone	7.26 (4.24)	6.94 (3.14)	6.37 (3.04)	0.51	0.95
Alanine	45.45 (1.92)	50.35 (1.79)	48.18 (1.75)	0.22	0.88
Ascorbate	17.15 (4.58)	18.08 (4.26)	16.06 (3.64)	0.76	0.99
Betaine	15.58 (2.88)	17.00 (3.13)	15.98 (2.95)	0.71	0.95
Carnitine	7.80 (2.37)	8.14 (2.60)	7.98 (2.49)	0.92	0.99
Choline	5.90 (1.79)	6.41 (1.66)	5.77 (1.74)	0.12	0.85
Citrate	332.14 (1.83)	370.58 (1.76)	330.72 (1.85)	0.16	0.85
Creatine	150.98 (4.29)	198.55 (3.79)	194.33 (3.65)	0.12	0.85
Dimethylamine	63.38 (1.52)	64.40 (1.46)	62.74 (1.47)	0.79	0.99
Ethanolamine	49.90 (1.40)	52.30 (1.42)	48.88 (1.39)	0.16	0.85
Formate	59.19 (2.43)	66.15 (2.36)	62.28 (2.40)	0.48	0.95
Fumarate	1.30 (2.18)	1.44 (2.39)	1.33 (2.13)	0.42	0.90
Glucose	50.55 (1.66)	51.84 (1.67)	51.61 (1.66)	0.86	0.99
Glycerol	32.93 (2.20)	37.52 (2.51)	36.06 (2.64)	0.35	0.89
Glycine	209.28 (1.92)	232.54 (1.79)	219.78 (1.89)	0.25	0.88
Hippurate	175.51 (2.31)	181.14 (2.32)	165.90 (2.16)	0.66	0.95
Histidine	122.05 (1.80)	133.17 (1.74)	129.45 (1.79)	0.28	0.88
Hypoxanthine	10.07 (2.01)	11.36 (1.94)	8.90 (2.10)	0.01	0.36
Isoleucine	2.22 (1.80)	2.23 (1.74)	2.17 (1.72)	0.90	0.99
Lactate	14.89 (2.48)	14.94 (2.04)	14.74 (2.59)	0.99	0.99
Leucine	3.84 (1.70)	3.84 (1.62)	3.83 (1.62)	1.00	0.99
Lysine	12.00 (2.31)	11.68 (2.00)	11.65 (2.19)	0.87	0.99
Methanol	5.47 (2.65)	5.90 (2.56)	5.46 (2.62)	0.67	0.95
O-Acetylcarnitine	4.02 (1.91)	4.33 (1.92)	3.91 (1.74)	0.34	0.89
Propylene.glycol	11.93 (4.15)	13.42 (4.66)	13.61 (4.32)	0.72	0.95
Quinolinate	6.58 (1.76)	6.60 (1.59)	6.09 (1.59)	0.30	0.88
Succinate	26.65 (2.43)	26.60 (2.52)	28.52 (2.35)	0.60	0.95
Taurine	78.23 (2.45)	78.46 (2.26)	79.58 (2.30)	0.98	0.99
Threonine	19.49 (1.76)	22.38 (1.75)	20.81 (1.68)	0.07	0.81
Trigonelline	9.68 (2.43)	8.47 (2.39)	10.11 (2.34)	0.14	0.85
Trimethylamine.N.oxide	35.68 (2.19)	38.28 (1.90)	32.01 (1.83)	0.07	0.81
Tryptophan	9.61 (1.62)	9.56 (1.66)	10.00 (1.58)	0.67	0.95
Valine	6.17 (1.69)	6.41 (1.66)	6.21 (1.62)	0.70	0.95
Cis-Aconitate	34.71 (1.53)	37.27 (1.55)	35.15 (1.53)	0.23	0.88
Π-Methylhistidine	25.31 (1.39)	26.01 (1.36)	25.05 (1.53)	0.61	0.95

*Unadjusted P-value

**P-value was adjusted for multiple comparisons using the Benjamini-Hochberg procedure

Table S2. Intraclass correlation for each metabolite

Metabolite	ICC	Within Subject Variance	Between Subject Variance
Succinate	0.528	0.416	0.371
Acetone	0.477	0.736	0.807
3.Aminoisobutyrate	0.470	0.348	0.393
Glucose	0.448	0.116	0.143
Isoleucine	0.444	0.14	0.175
Betaine	0.442	0.528	0.667
Lysine	0.437	0.26	0.335
Valine	0.436	0.11	0.143
Histidine	0.434	0.144	0.188
Choline	0.423	0.128	0.174
Dimethylamine	0.421	0.065	0.089

Tryptophan	0.418	0.097	0.135
Alanine	0.413	0.149	0.212
Acetate	0.396	0.411	0.628
Fumarate	0.396	0.256	0.39
Leucine	0.377	0.093	0.154
4.Hydroxyphenylacetate	0.377	0.096	0.159
3.Hydroxyisovalerate	0.375	0.108	0.179
Glycine	0.365	0.142	0.248
Methanol	0.354	0.326	0.595
cis.Aconitate	0.348	0.064	0.121
Formate	0.333	0.254	0.509
Lactate	0.333	0.248	0.497
4.Hydroxybenzoate	0.332	0.158	0.317
1.Methylnicotinamide	0.323	0.153	0.32
2.Aminobutyrate	0.321	0.234	0.495
2.Oxoglutarate	0.319	0.167	0.357
3.Hydroxybutyrate	0.319	0.163	0.348
Glycerol	0.318	0.256	0.55
Ethanolamine	0.305	0.035	0.079
2.Hydroxybutyrate	0.303	0.133	0.306
Creatine	0.29	0.541	1.324
3.Indoxylsulfate	0.284	0.086	0.217
?-Methylhistidine	0.278	0.035	0.092
Threonine	0.272	0.082	0.22
Trigonelline	0.263	0.2	0.559
Quinolinate	0.252	0.063	0.187
Taurine	0.243	0.174	0.542
Citrate	0.242	0.086	0.27
Trimethylamine.N.oxide	0.237	0.111	0.358
Ascorbate	0.23	0.464	1.557
Hypoxanthine	0.22	0.11	0.391
Carnitine	0.195	0.161	0.665
O.Acetylcarnitine	0.188	0.072	0.311
2.Hydroxyisovalerate	0.178	0.052	0.242
Acetoacetate	0.155	0.065	0.354
Propylene.glycol	0.144	0.314	1.859
Hippurate	0.122	0.081	0.585

Table S3. P-values for the likelihood ratio test for the full adjusted model compared with the null models.

Metabolite	KEGG ID	Unadjusted p value	Benjamini-Hochberg adjusted p-values

1-Methylnicotinamide	C02918	0.02	0.52
3-Hydroxybutyrate	C01089	0.03	0.52
Trimethylamine-N-oxide (TMAO)	C01104	0.04	0.52
Citrate	C00158	0.06	0.52
Quinolinate	C03722	0.06	0.52
Betaine	C00719	0.07	0.52
Histidine	C00135	0.11	0.65
3-Indoxylsulfate	N/A	0.13	0.65
Glycine	C00037	0.15	0.65
Ethanolamine	C00189	0.19	0.65
Hippurate	C01586	0.2	0.65
Cis-Aconitate	C00417	0.2	0.65
Choline	C00114	0.21	0.65
τ-Methylhistidine	C01152	0.21	0.65
Dimethylamine	C00543	0.22	0.65
2-Oxoglutarate	C00026	0.23	0.65
2-Hydroxyisovalerate	N/A	0.24	0.65
Isoleucine	C00407	0.25	0.65
Methanol	C00132	0.27	0.65
4-Hydroxybenzoate	C00156	0.28	0.65
Formate	C00058	0.28	0.65
Carnitine	C00318	0.3	0.65
Acetoacetate	C00164	0.32	0.65
Hypoxanthine	C00262	0.32	0.65
O-Acetylcarnitine	C02571	0.35	0.66
Tryptophan	C00078	0.37	0.66
2-Aminobutyrate	C02356	0.38	0.66
Lysine	C00047	0.39	0.66
Ascorbate	C00072	0.4	0.66
Fumarate	C00122	0.43	0.66
Glucose	C00031	0.44	0.66
Glycerol	C00116	0.44	0.66
Trigonelline	C01004	0.49	0.71

Lactate	C00186	0.5	0.71
3-Hydroxyisovalerate	N/A	0.53	0.73
Valine	C00183	0.54	0.73
Creatine	C00300	0.61	0.78
Acetate	C00033	0.62	0.78
Succinate	C00042	0.64	0.78
Alanine	C00041	0.65	0.78
Propylene-glycol	C00583	0.68	0.79
2-Hydroxybutyrate	C05984	0.69	0.79
Acetone	C00207	0.73	0.80
Leucine	C00123	0.74	0.80
3-Aminoisobutyrate	C05145	0.75	0.80
Threonine	C00188	0.77	0.81
Taurine	C00245	0.87	0.88
4-Hydroxyphenylacetate	C00642	0.88	0.88
